# Smartphone Application-Enabled Apple Fruit Surface Temperature Monitoring Tool for In-Field and Real-Time Sunburn Susceptibility Prediction

**DOI:** 10.3390/s20030608

**Published:** 2020-01-22

**Authors:** Bin Wang, Rakesh Ranjan, Lav R. Khot, R. Troy Peters

**Affiliations:** 1 College of Information Science and Technology, Hebei Agricultural University, Baoding 071001, China; binwang@hebau.edu.cn; 2 Department of Biological Systems Engineering, Center for Precision and Automated Agricultural Systems, Washington State University, Prosser, WA 99350, USA; rakesh.ranjan@wsu.edu (R.R.); troy_peters@wsu.edu (R.T.P.)

**Keywords:** crop loss management, apple sunburn prediction, fruit surface temperature, thermal-RGB image, smartphone application, Android OS, ‘AppSense 1.0’

## Abstract

Heat stress and resulting sunburn is a major abiotic stress in perineal specialty crops. For example, such stress to the maturing fruits on apple tree canopies can cause several physiological disorders that result in considerable crop losses and reduced marketability of the produce. Thus, there is a critical technological need to effectively monitor the abiotic stress under field conditions for timely actuation of remedial measures. Fruit surface temperature (FST) is one of the stress indicators that can reliably be used to predict apple fruit sunburn susceptibility. This study was therefore focused on development and in-field testing of a mobile FST monitoring tool that can be used for real-time crop stress monitoring. The tool integrates a smartphone connected thermal-Red-Green-Blue (RGB) imaging sensor and a custom developed application (‘AppSense 1.0’) for apple fruit sunburn prediction. This tool is configured to acquire and analyze imagery data onboard the smartphone to estimate FST. The tool also utilizes geolocation-specific weather data to estimate weather-based FST using an energy balance modeling approach. The ‘AppSense 1.0’ application, developed to work in the Android operating system, allows visual display, annotation and real-time sharing of the imagery, weather data and pertinent FST estimates. The developed tool was evaluated in orchard conditions during the 2019 crop production season on the Gala, Fuji, Red delicious and Honeycrisp apple cultivars. Overall, results showed no significant difference (*t_110_* = 0.51, *p* = 0.6) between the mobile FST monitoring tool outputs, and ground truth FST data collected using a thermal probe which had accuracy of ±0.4 °C. Upon further refinements, such tool could aid growers in real-time apple fruit sunburn susceptibility prediction and assist in more effective actuation of apple fruit sunburn preventative measures. This tool also has the potential to be customized for in-field monitoring of the heat stressors in some of the sun-exposed perennial and annual specialty crops at produce maturation.

## 1. Introduction

Sunburn is a major physiological disorder that adversely affects productivity of fruits and vegetables including apple, grapes, pepper, pumpkin and watermelons [1,2,3,4,5]. Fruit sunburn in apples has been reported in all major apple growing regions around the globe and causes 10–40% yield losses. Sunburn is caused by sunlight with high intensity, ultra-violet (UV) radiation and photo-synthetically active radiation (PAR) that elevates maturing fruit peel temperature during the field production. Sunburn necrosis, sunburn browning and photo-oxidative sunburn are the three major sunburn types [4,5]. Sunburn necrosis leads to thermal death of epidermal and sub-epidermal cells (peel) and takes place when fruit surface temperature (FST), a reliable indicator of sunburn, reaches 52 ± 1 °C. Sunburn browning occurs at a slightly lower FST, i.e., between 46 and 49 °C, whereas the photo oxidative sunburn occurs at a much lower FST because of PAR and light shocks to sudden sun-exposure of shaded apple due to management practices such as hand thinning, selective picking or pruning operations [2,3,4,5,6,7,8,9]. Apart from sunburn, FST also influences several physiological and biological processes in fruit such as flavor and nutritional values, sugar and acid content, ripening process, fruit quality, size, appearance, and insect/pest infestation susceptibility.

Fruit sunburn forecasting has been conventionally done using the air temperature data collected by open field weather stations. Often, such an approach is highly unreliable and can result in untimely application of water for evaporative cooling of canopies and fruits. Excess evaporative cooling can be water and energy inefficient and may create food safety issues. A weather-data-based apple surface-temperature dynamics model [10] that uses parameters such as wind speed, humidity, solar intensity, and dew point temperature, apart from air temperature, could be useful for FST estimates. However, it requires on-site sensors to get reliable micro-climate weather data. Thermocouples, thermometers and thermal probes are also used for FST estimation. However, such probes installation tends to be laborious, less accurate, data limited and destructive; limiting wide scale adaptation.

In recent years, portable miniaturized thermal imaging sensors have been explored in various crop-stress monitoring applications including that of rapid apple FST monitoring [10]. During field use, thermal imagery can be inadequate as it can have challenges in segmenting regions of interests (fruits) within the background branches, stems and leaves [11]. However, when combined with RGB imagery data, thermal imagery can be used for apple fruit surface temperature monitoring [11]. Our team has developed and successfully tested such smart apple sunburn sensing systems [12]. Nevertheless, these orchard sensing system needs to be rugged, economically viable, and connected to a reliable cellular network that may not be available at many orchards. These challenges with existing systems raise a need for a user friendly, low cost, and portable sunburn prediction tool that will aid growers in real-time, accurate, apple sunburn monitoring and pertinent decision-making.

Our team has envisioned such a mobile FST monitoring tool that leverages advances in low cost commercial thermal-RGB imaging sensors and smartphone technologies that allow ease of integration. Smartphones are becoming commonly available to wider demographics and have opened a new horizon for smartphone-based applications that helps in solving daily-life problems. Recently, researchers have taken advantage of the increased computing potential, accessibility, ease-of-operation and low cost of smartphones for developing pertinent use cases in medical diagnosis, environmental monitoring and food safety [13]. Related to agriculture applications, Wang et al. [14] utilized the smartphone built-in camera and developed an application for in-field fruit size estimation. Das et al. [15] has developed a prototype for a smartphone-based spectrometer for rapid and non-destructive fruit ripeness testing.

Overall, smartphone-based applications integrated with compatible imaging and optical sensors have a huge potential for effective orchard crop stress monitoring. Therefore, this study focused on the development of a smartphone-enabled, thermal-RGB, and microclimate-sensing assisted, real-time apple sunburn prediction tool. The specific study objectives were to:

(1) Develop a smartphone enabled miniaturized and portable apple fruit surface temperature monitoring tool with a compatible application (‘AppSense 1.0’) for real-time fruit surface temperature estimation.
(2) Evaluate the performance of the developed tool in orchard conditions towards predicting sunburn susceptibility of four fresh market apple cultivars *cv.* ‘Gala’, ‘Fuji’, ‘Red delicious’ and ‘Honeycrisp’.

## 2. Materials and Methods

### 2.1. Mobile Fruit Surface Temperature Monitoring Tool

**Figure 1:**
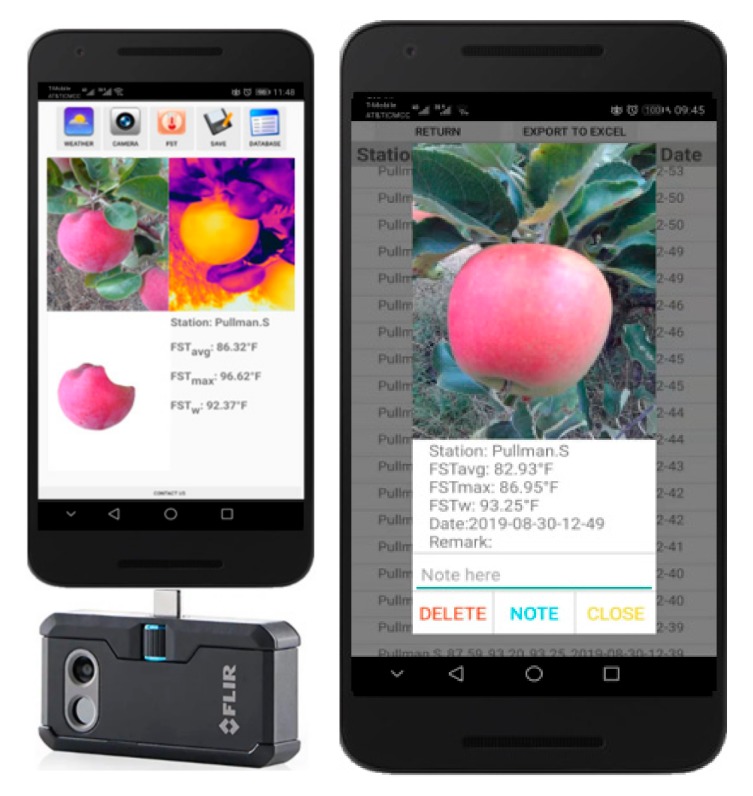
Mobile fruit surface temperature monitoring tool with ‘AppSense 1.0’ Android application designed for rapid and real-time apple sunburn prediction.

The mobile FST monitoring tool (Figure 1) consists of a thermal-RGB sensor (FLIR One Pro, FLIR Systems, Inc., Wilsonville, Oregon, OR, USA) integrated with an Android operating system (OS) based smartphone. Typical design consideration was that such a tool should work on any smartphone that has on-the-go universal serial bus [OTG USB] and ‘Android 8.0’ or higher OS version. This tool uses an Android OS compatible application (version: 1.0; hereafter termed as ‘AppSense 1.0’) that allows data acquisition, processing, management and sharing with the end-users. The mobile FST tool enables real-time acquisition of in-field thermal-RGB images and geolocalized weather data for resulting data analysis on-board the smartphone to estimate apple FST. The imagery and weather-data-based FST estimates can be stored on board the smartphone and shared with end-users to make appropriate sunburn management related decisions. This tool was designed in such a way that a typical fruit grower or farm crew can install and use it with ease.

### 2.2. Smartphone Application Development

**Figure 2:**
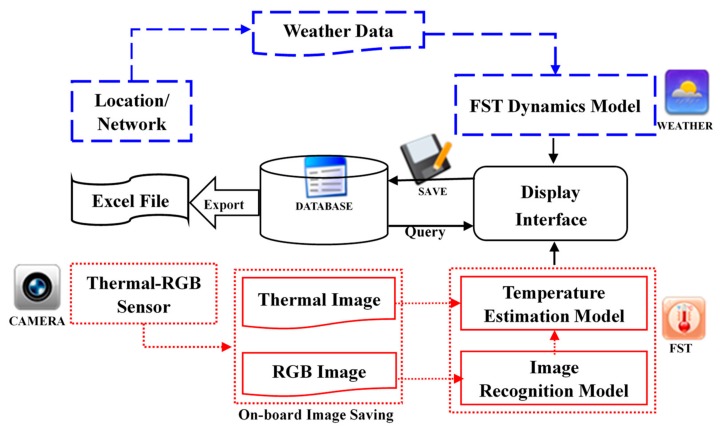
The ‘AppSense 1.0’ application data analysis flow (blue and red lines represent the weather data based fruit surface temperature (FST) and imaging based FST models, respectively). Indicated icons on the work-flow represent various controls (CAMERA-image acquisition; WEATHER-weather data collection form nearest weather station; FST-estimation of imagery and weather based FST; SAVE-save the data on-board the smartphone, DATABASE-summarize the result and exports to ‘*.xlse’ file).

The ‘AppSense 1.0’ application was developed in ‘Android Studio’ integrated development environment (IDE) (open source, version 3.4.2). This application was designed to collect weather data from the nearest open field weather station, acquire the thermal-RGB image, estimate the weather and imagery based FSTs, save and display the processed images and FST data, and export them in excel format (‘*.xlxs’) (Figure 2). An open source computer vision (OpenCV, version 3.4.0) Java library was utilized for on-board image processing and FST estimation. The ‘AppSense 1.0’-captured raw and processed images were stored in the internal memory of the smartphone that can be utilized for further analysis. The data analyzed by the tool is stored in the local ‘SQLite’ database and can be retrieved and managed at a later stage.

### 2.3. Weather Data Based FST Estimation

An apple surface temperature dynamics model by Li et al. [10] was utilized for weather data based FST estimation. The energy balance approach was adapted for the model development. The total input energy in the fruit system that includes net short-wave radiation and net long wave radiation was balanced with total output energy, that includes emitted fruit thermal radiation, energy loss by evapotranspiration, sensible heat loss or gain, and total heat transfer within the fruit. The derived model utilizes four major weather parameters (air temperature, dew point temperature, solar radiation and wind speed) and the surrounding ground temperature to predict the mean apple FST. The open field weather data was acquired in real time from the agricultural weather network of Washington State University (WSU AgWeatherNet, available online: http://weather.wsu.edu/index.php). This network includes 186 automated weather stations located mostly in the irrigated regions of eastern Washington State [16]. Typically, each station collects 10 different weather parameters at a frequency of 0.2 Hz and provide mean value at every 15 min.

**Figure 3:**
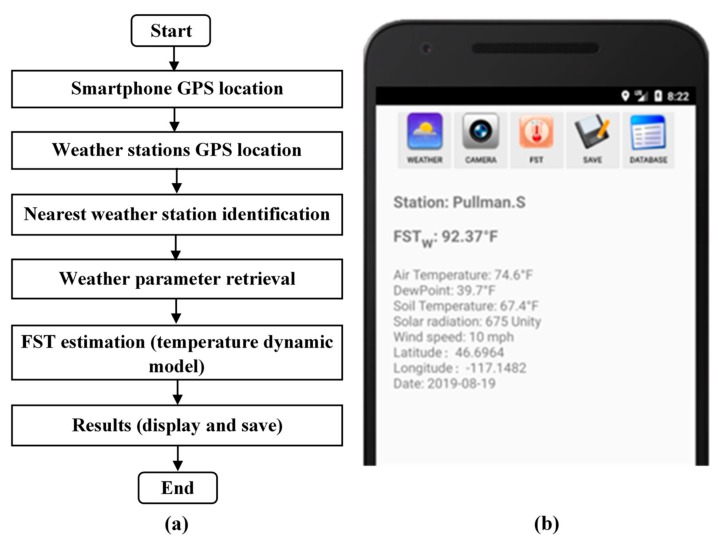
Weather data based (**a**) FST estimation process flow and (**b**) location specific weather information and FST estimation results.

The process flow of weather based FST estimation is depicted in Figure 3. Firstly, the ‘AppSense 1.0’ application acquires longitude and latitude information of all weather stations in the network and utilizes the smartphone global positioning system (GPS) coordinates (longitude and latitude) to locate the nearest weather station. The FST monitoring tool downloads the weather parameters from the located station and applies the apple surface temperature dynamics model to estimate the FST.

### 2.4. Thermal-RGB Imagery Based FST Estimation

**Figure 4:**
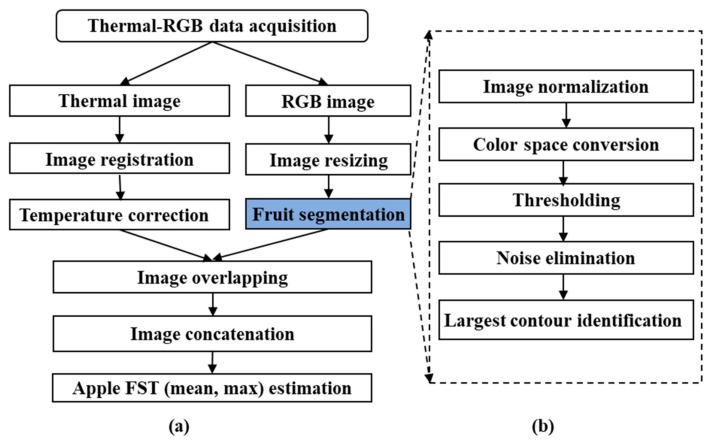
(**a**) Flow chart for thermal-Red-Green-Blue (RGB) imaging based FST estimation and (**b**) steps involved in color feature based fruit segmentation.

A customized image-processing algorithm was developed for estimating thermal-RGB imagery data based FST (Figure 4). The smartphone-integrated thermal-RGB imaging sensor (measurement range: −20–400 ℃, Accuracy: ±3 ℃ or ±5%, focal length: 15 cm–infinity, thermal sensitivity: 70 mK) was capable of acquiring radiometric thermal (resolution: 160 × 120 pixels) and Red-Green-Blue (RGB) (resolution: 1440 × 1080 pixels) images (Figure 4). Radiometric thermal data consists of pixelated temperature information.

Calibration of the thermal-RGB sensor was conducted using water [17] with an emissivity of 0.95–0.97, almost equal to that of plant leaves and apples surfaces. The water temperature was varied within a range of 10–50 °C in reference to typical FSTs. The actual temperature was measured using a liquid-in-glass thermometer for calibration of the thermal-RGB sensor-estimated temperatures. Five random circular spots were selected from the images and were analyzed for their mean temperatures in the sensor manufacturer provided Application (FLIR One, FLIR Systems Inc., Wilsonville, Oregon, OR, USA). The developed calibration equations were then used to correct the temperature data.

In this study, the pertinent RGB imagery data was used to identify and segment the apple from the rest of the canopy. The size of the RBG image was first resized to 160 × 120 pixels so that it could be overlapped with the thermal images for accurate pixel matching. Fruit color feature was used to separate the fruit from the stems, foliage, and background. For simplicity, the fruit shape feature extraction method was not employed as it is expected that the user will point the sensor to have several fruits prominent in the image.

#### 2.4.1. Color Space Conversion and Fruit Segmentation

The *YUV* color space is one of the closest to the color perception of human eyes. The use of the *YUV* space may be more effective than *RGB* color space [18]. In *YUV*, Y represents the brightness of color space and can be very vulnerable to the influence of illumination. Hence, only *U* (red projection) and *V* (blue projection) color components were used for segmentation. Moreover, such color conversion makes the algorithm simple from a computational resource use point of view [19]. The *RGB* to *YUV* color transformation algorithm have been illustrated in Equation (1):

(1) $$\left(\begin{array}{c} Y\\ U\\ V \end{array}\right)=\left(\begin{array}{ccc} 0.299 & 0.587 & 0.114\\ -0.14713 & -0.2886 & 0.436\\ 0.615 & -0.51499 & -0.10001 \end{array}\right)\left(\begin{array}{c} R\\ G\\ B \end{array}\right)$$

The resulting *YUV* color space was then used to segment the fruit using the logic in Equation (2).

if (U>U1 && U<U2 && V>V1 && V<V2)(2)

RGB pixel value = 255

else

RGB pixel value = 0

where, *U* and *V* is the chrominance component of the *YUV* color space, *U*1 and *V*1 are the lower threshold, and *U*2 and *V*2 are the upper threshold of *U* and *V*, respectively. The threshold was set based on image analysis of more than a hundred mature apples of selected cultivars. First, the apples were manually segmented using an image-editing tool (Photoshop CS6, Adobe Inc., San Jose, California, CA, USA). The mean *YUV* values of each of the segmented apples were then investigated and the upper and lower threshold of the chrominance components were determined. The resulting *U*1 and *U*2 values were set at −0.07 and 0.18, and the *V*1 and *V*2 values were set at −0.12 and 0.58, respectively.

**Figure 5:**
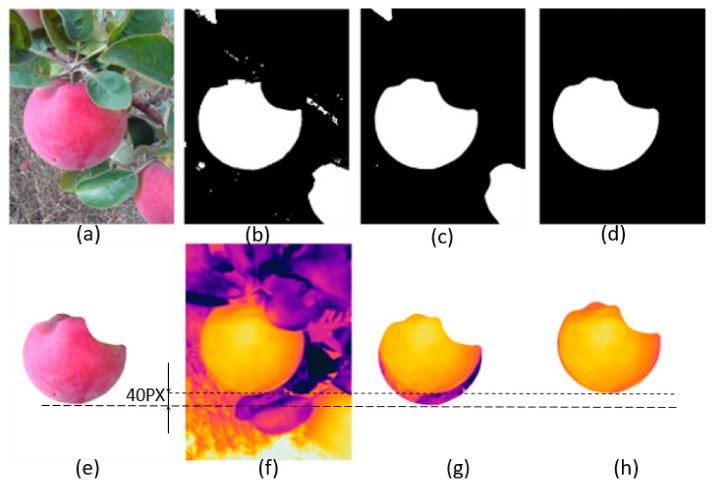
A typical thermal-RGB image analysis outputs (**a**) an RGB image, (**b**) a segmented and binarized image, (**c**) a binary image after blur filtering, (**d**) a binary image with the largest contour, (**e**) an apple blob extraction, (**f**) a raw thermal image, (**g**) a pixel offset between the raw overlapped thermal and RGB images, and (**h**) an offset-adjusted image overlay.

Most of the segmented binary images (Figure 5b) had residual errors. Branches, stems and leaves in the frame were sometimes closer to the color features of maturing apples and can lead to such errors. A median Blur filter (size = 25 pixels), was applied to remove these errors (Figure 5c). This filter eliminated the small unwanted pixels from the frame. However, since only the largest apple in the image was the region of the interest for this study, the use of a fixed filter size was not completely effective and some parts of partially segmented apples remained in the region of the interest. To eliminate such areas, the maximum connected pixel domain was identified and the rest were rejected (Figure 5d). Figure 5e depicts the resulting segmented apple blob. Finally, the segmentation accuracy of 10 randomly selected sample images were estimated. The largest apple in the frame was manually labeled for each image and the segmentation accuracy of the algorithm was evaluated by dividing the area of the algorithm-segmented image with that of the manually segmented ground-truth image at each stage.

#### 2.4.2. Image Overlapping and FST Estimation

A pixel offset was observed between the thermal and RGB image (Figure 5e,f). The eccentricity between two sensors often results in such an offset. The pixel offset was found to be about 40 pixels when the image was captured from a typical imaging distance of 20 cm. An image registration was performed between the thermal and RGB images to eliminate this offset. Finally, arithmetic operations were performed on the segmented thermal image (Figure 5h) to estimate the mean and maximum apple FST.

### 2.5. Field Tests

**Figure 6:**
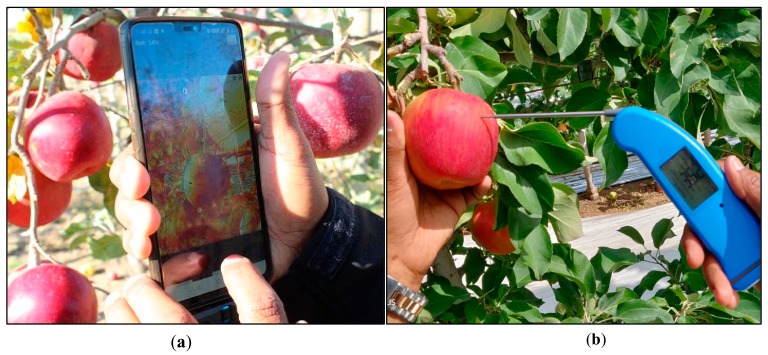
(**a**) Fruit surface temperature monitoring using developed tool and pertinent (**b**) ground-truthing using a thermal probe.

Field data was collected for four apple cultivars *cv.* Honeycrisp, Fuji, Gala and Red delicious. The orchards were at various locations within the state of Washington, USA (Table 1). First, 60 images were captured from an imaging distance of about 20 cm for *cv.* Honeycrisp using thermal-RGB imaging sensor integrated with an Android OS smartphone (model: OnePlus 6, OnePlus Technology Co. Ltd., Shenzhen, China) (Figure 6a). The image acquisition and on-board processing was performed using the ‘AppSense 1.0’ application to estimate FST. Moreover, actual surface temperature, or ground truth data was collected using a thermal probe (model: Thermapen^®^ Mk4, ThermoWorks, Lindon, Utah, UT, USA) of accuracy ±0.4 °C and response time of 2–3 s. Five sun-exposed apples were randomly selected in the orchard block and the thermal probe was punched into the upper 3 mm of the apple surface at five places to measure the surface temperature (Figure 6b). A stabilization time of 3 s was used for reliable temperature measurement. This data was used to test and refine the mobile FST monitoring tool and the functionality of the developed application. The resulting tool was further tested to monitor the FST of *cv.* Fuji (50 fruits), Gala (75 fruits) and Red delicious apples (20 fruits).

### 2.6. Data Analysis

The thermal-RGB imagery derived FST (FST_i_), weather data derived FST (FST_w_), ground truth measured FST (FST_G_), and ambient air temperature (T_air_) data was first checked for normality as a primary step in statistical analysis. A ‘Pearson product moment’ correlation test was then performed to test the relationship between FST_i_, FST_G,_ and T_air_. Moreover, a ‘two-sample t-test’ was performed to quantify the mean difference between FST_i_ and FST_w_. A similar analysis was performed to estimate the mean difference between FST_i_ and FST_G_, FST_i_ and T_air_, as well as imagery based maximum FST (FST_i-max_) and FST_G_. A statistical analysis was performed in R-Studio^®^ (version 1.0.153–© 2009–2017, RStudio^®^, Boston, MA, USA) and significance was inferred at the 5% level.

## 3. Results and Discussion

### 3.1. Fruit Segmentation

**Figure 7:**
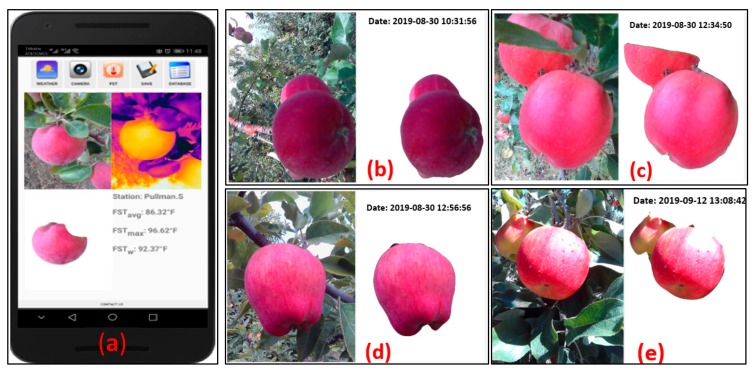
(**a**) The ‘AppSense 1.0’ application display interface depicting segmentation output and summary of FST estimates and representative segmentation results obtained for (**b**) Fuji, (**c**) Gala, (**d**) Red delicious, and (**e**) Honeycrisp fruits through real-time imagery data analysis on-board the smartphone running the mobile FST monitoring tool.

Figure 7 depicts the representative segmentation results obtained for all four fresh market apple cultivars tested in this study. Within the ‘AppSense 1.0’ ecosystem, the developed algorithm provided a mean segmentation accuracy of 94.4%, 95.7%, and 97.5% at respective thresholding, blur filtering, and final apple blob extraction stages (Table 2). The algorithm was able to segment matured apples from the background canopy attributes. Results indicated that different apple cultivars have some effect on the segmentation algorithm accuracy. The algorithm performed best for the *cv.* Red delicious as the dark and intense crimson color of fruit makes it less reflective and easier to distinguish from surrounding objects. Furthermore, the number of apples in the frame had no visual effect on the segmentation as only the maximum connected pixel domain was selected and the rest of the apples in the frame were filtered out. However, the apple glossiness that can produce a bright white spot on apples during imaging had an adverse effect on the segmentation due to color illusion (Figure 7e). Appropriate angle of the imaging sensor can help in avoiding these bright spots for accurate segmentation. Moreover, unsupervised classification-based techniques [12] and supervised machine learning techniques could be further explored for robust segmentation and are the subject of our on-going research.

### 3.2. Apple Fruit Surface Temperature

#### 3.2.1. Reliability of the Mobile FST Monitoring Tool

**Figure 8:**
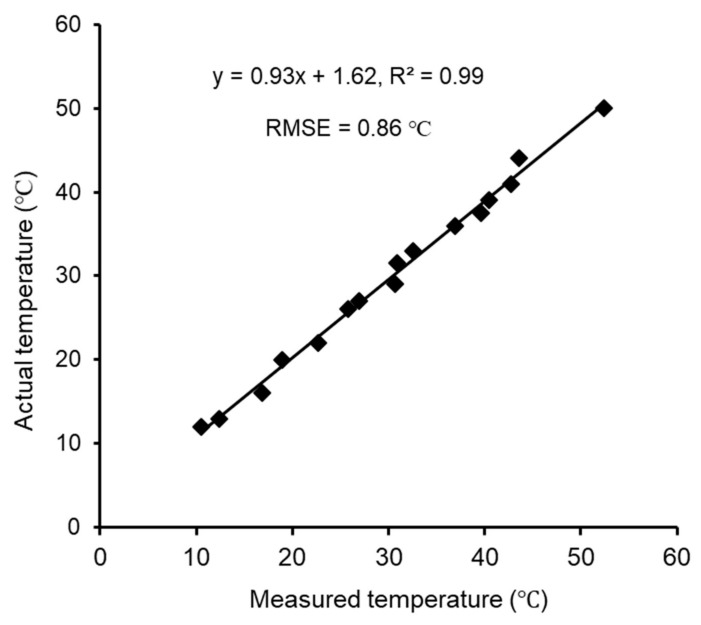
Thermal-RGB imaging sensor calibration equation for temperature data correction.

**Figure 9:**
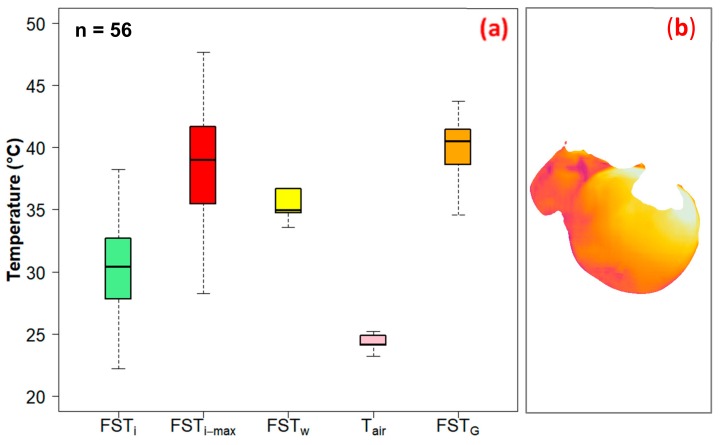
(**a**) Thermal-RGB imagery and weather data derived FST estimates and corresponding ground truth measured FST and ambient air temperature data (FST_i_: Thermal-RGB imagery derived FST, FST_i-max_: imagery based maximum FST, FST_w_: weather data derived FST, T_air_: ambient air temperature, FST_G_: ground truth measured FST, and n: sample size), and (**b**) typical fruit thermal map for *cv.* Honeycrisp.

The calibration results showed a strong correlation (R^2^ = 0.99) and low measurement error (RMSE = 0.86 ℃) (Figure 8) between actual temperature and the thermal-RGB sensor measured temperature data. The statistical analysis of FST_i_ and FST_G_ for *cv.* Honeycrisp indicated that there was no significant difference in FST_i-max_ (mean ± standard deviation) (38.8 ± 3.7 ℃) and FST_G_ (39.3 ± 3.8 ℃) (Two sample *t*-test, *t*_110_ = 0.51, *p* = 0.6) (Figure 9). Ground truth measurements were taken at the upper neck area of the apples, as it was observed to be the hottest area on the fruit and therefore the most susceptible to sunburn. The close association of FST_i-max_ and FST_G_ suggest that the developed tool is reliable for FST monitoring in orchard conditions. There was a significant difference in mean FST_i_ (30.4 ± 2.9 ℃) and FST_G_ (*t*_110_ = 12.5, *p* < 0.0001). This is arguably because FST_i_ was a mean FST value of overall segmented apple whereas ground truth measurement targeted the hottest point of the apple.

#### 3.2.2. Field Evaluation of the Mobile FST Monitoring Tool

A significant temperature difference was reported between the weather parameter derived mean FST (FST_w_) (35.3 ± 3.4 ℃) and the imagery data derived mean FST_i_ (*t*_110_ = −9.61, *p* < 0.0001) for *cv.* Honeycrisp (Figure 9). The ‘AppSense 1.0’ application used data from an open field weather station that was located in the ranges of 4 to 7 km from the orchard sites (Table 1). The weather parameters from the closest identified weather stations might have large differences from the local microclimate in the test orchard sites. Such microclimate variation might have resulted in the FST_w_ and FST_i_ differences. A site-specific in-field weather station could assist in eliminating such errors and help estimate more accurate weather-parameter derived FSTs. Ongoing research in our lab is focused on in-field microclimate parameter-based FST estimation.

**Figure 10:**
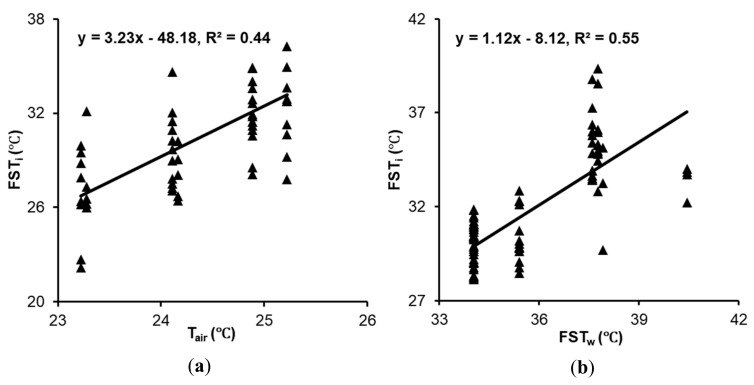
Relationships between FST_i_ and (**a**) FST_w_ and (**b**) T_air_ (FST_i_: Thermal-RGB imagery derived FST, FST_w_: weather data derived FST, T_air_: ambient air temperature).

The FST_i_ and air temperature (T_air_) was significantly correlated with each other (Pearson product moment correlation, *r* = 0.66, *t*_54_ = 6.4, *p* < 0.001) (Figure 10a). While comparing the FST and ambient air temperature (T_air_), the mean FST_i_ and FST_w_ was significantly higher than T_air_ (24.4 ± 2.3 ℃). Racsko and Schrader [3] and Shi et al. [12] have also reported a temperature difference of 5–18 ℃ between air temperature and apple FST estimates.

**Figure 11:**
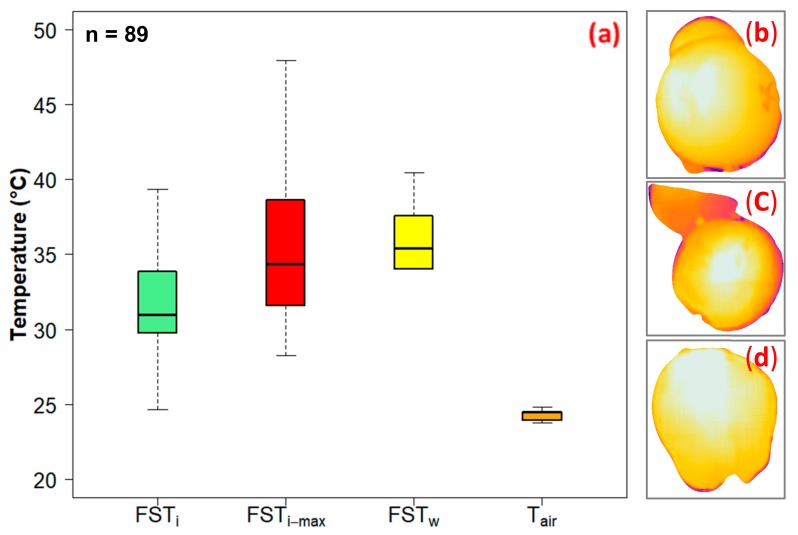
(**a**) Thermal RGB imagery and weather data derived FST estimates and corresponding ambient air temperature with temperature map for *cv.* (**b**) Fuji, (**c**) Gala and (**d**) Red delicious (FST_i_: Thermal-RGB imagery derived FST, FST_i-max_: imagery based maximum FST, FST_w_: weather data derived FST, T_air_: ambient air temperature, FST_G_: ground truth measured FST, and n: sample size).

Analysis indicated a similar FST trend for *cv.* Fuji, Gala and Red delicious. A significant correlation was reported between FST_w_ and FST_i_ (*r* = 0.74, *t*_86_ = 10.4, *p* < 0.001) (Figure 10b). Moreover, there was no significant difference between FST_i-max_ (35.6 ± 2.4 ℃) and FST_w_ (35.7 ± 2.7 ℃) (*t*_174_ = −0.2, *p* = 0.84) (Figure 11). However, there was a significant difference in FST_w_ and mean FST_i_ (31.8 ± 2.4 ℃) (*t*_174_ = −10.7, *p* < 0.0001). Further investigation indicates that the mean FST_i_ and FST_w_ for *cv.* Fuji, Gala and Red delicious was significantly higher than T*_air_* (24.3 ± 1.8 ℃).

## 4. Conclusions

Followings are the conclusions from this study:

1. The mobile FST monitoring tool performed reliably in field conditions to estimate FSTs of the four most common apple cultivars grown in the state of Washington. The developed smartphone application ‘AppSense 1.0‘, successfully acquired thermal-RGB images and weather data and was able to perform real-time data analysis on-board the smartphone to estimate apple FSTs.
2. The mobile FST monitoring tool measured maximum FST was consistent with ground truth measurements. A significant difference was recorded between weather and thermal-RGB imagery predicted FSTs, possibly due to the remote locations of the open field weather stations that were 4–7 km away from the experimental sites.

Overall, with further refinements and application deployment, the developed mobile sunburn prediction tool is expected to aid growers in accurate and real-time fruit surface temperature monitoring and actuation of subsequent control measures to avoid sunburn related crop losses. Such tools can also assist in monitoring other FST-influenced physiological parameters such as sugar and acid content, ripening process, fruit color and quality. Moreover, this tool has the potential to be customized for monitoring heat stressors in some of the sun-exposed perennial and annual specialty crops at produce maturation in the field.

## Figures and Tables

**Table 1:** Details of field data collection using the developed mobile FST monitoring tool at various orchard locations within the state of Washington, USA.

Cultivar	Number of Images	Latitude/Longitude (N, W)	Nearby Town	Distance from Nearest Weather Station, km
Honeycrisp	60	46.6249, −120.6187	Yakima	7.35
Fuji	50	46.7324, −117.1261	Pullman	4.14
Gala	40	46.6964, −117.1482	Pullman	4.14
Red delicious	20	46.7331, −117.1264	Pullman	4.14
Gala	35	46.2830, −119.6379	Benton city	4.33

**Table 2:** Segmentation accuracy of the algorithm at various processing steps.

Image	Segmentation Accuracy (%)		
	Threshold Image	Blur Filtering	Extracted Apple Blob
1	91.4	92.2	98.9
2	91.6	93.8	97.1
3	95.6	97.3	98.2
4	94.9	96.4	97.8
5	97.4	97.9	98.5
6	93.1	94.7	96.5
7	94.1	95.1	97.6
8	95.7	96.8	97.3
9	94.6	96.3	96.7
10	96.0	96.2	96.7
Mean	94.4	95.7	97.5
Standard deviation	1.6	1.6	0.8
